# The DNA Glycosylases Ogg1 and Nth1 Do Not Contribute to Ig Class Switching in Activated Mouse Splenic B Cells

**DOI:** 10.1371/journal.pone.0036061

**Published:** 2012-04-20

**Authors:** Anna J. Ucher, Erin K. Linehan, George W. Teebor, Carol E. Schrader, Janet Stavnezer

**Affiliations:** 1 Department of Microbiology and Physiological Systems, Medical School, University of Massachusetts, Worcester, Massachusetts, United States of America; 2 Department of Pathology, Kaplan Comprehensive Cancer Center, New York University Medical Center, New York, New York, United States of America; Universita' di Milano, Italy

## Abstract

During activation of B cells to undergo class switching, B cell metabolism is increased, and levels of reactive oxygen species (ROS) are increased. ROS can oxidize DNA bases resulting in substrates for the DNA glycosylases Ogg1 and Nth1. Ogg1 and Nth1 excise oxidized bases, and nick the resulting abasic sites, forming single-strand DNA breaks (SSBs) as intermediates during the repair process. In this study, we asked whether splenic B cells from mice deficient in these two enzymes would show altered class switching and decreased DNA breaks in comparison with wild-type mice. As the *c-myc* gene frequently recombines with the IgH S region in B cells induced to undergo class switching, we also analyzed the effect of deletion of these two glycosylases on DSBs in the *c-myc* gene. We did not detect a reduction in S region or *c-myc* DSBs or in class switching in splenic B cells from Ogg1- or Nth1-deficient mice or from mice deficient in both enzymes.

## Introduction

Immunoglobulin (Ig) class switching occurs in activated mature B cells after immunization or infection in vivo, and causes an exchange of the IgM isotype for IgG, IgE or IgA isotypes, resulting in increased effectiveness of the humoral immune response. Class switching occurs by a DNA recombination event, termed class switch recombination (CSR), which can be induced in cultured mouse splenic B cells by treatment with bacterial lipopolysaccharide (LPS). CSR occurs via a mechanism that involves induction of double-strand DNA breaks (DSBs) in special switch (S) region sequences located upstream of each isotype gene, followed by non-homologous end joining between donor and acceptor S regions. Activation-induced cytidine deaminase (AID) initiates DSB formation in S regions by deamination of cytosines, converting them to uracils. Then DSBs are created by the action of both the base excision repair pathway and by mismatch repair [1]. Although AID primarily targets Ig genes, it also deaminates other sites in the genome, and this can lead to mutations, DNA breaks, and to translocations [2], [3], [4], [5], [6]. In fact, translocations between the *c-myc* gene and the IgH locus are a relatively frequent event in mouse B cells induced with LPS, and also occur in vivo and lead to lymphomagenesis.

During B cell activation in culture by LPS, large amounts of reactive oxygen species (ROS) are generated [7], [8], [9]. High levels of ROS can result in oxidized DNA bases, which like AID-induced dUs, are also repaired by base excision repair (BER), a pathway that results in single-strand DNA breaks (SSBs) as intermediates. Ogg1 and Nth1 are two BER glycosylases that perform both the initial excision of the oxidized base and the incision of the DNA backbone during the process of removing oxidized guanine (8-oxo-G) and oxidized pyrimidines (cytosine glycol and thymine glycol), respectively [10], [11]. We hypothesized that if the increased ROS produced during LPS-activation of B cells were to result in oxidized dG and pyrimidines, then Ogg1 and Nth1 might contribute to formation of DNA breaks in S regions or in the *c-myc* locus in these cells. To test this hypothesis, we determined whether deficiencies in Ogg1 and/or Nth1 would lead to decreased DNA DSBs in Sμ or *c-myc*. We also examined the efficiency of CSR, because if these Sμ DSBs were important for CSR, then CSR might be decreased in B cells deficient in these enzymes.

## Results

**Figure 1:**
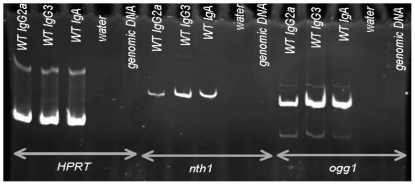
RT-PCR for Ogg1 and Nth1 mRNA demonstrates that these genes are expressed in activated WT splenic B cells. Splenic B cells were treated for 2 days as described in Methods in order to induce CSR to IgG3, IgG2a, or IgA. The lanes are labeled as to the isotype to which the cells were induced to switch. Lanes labeled “water" have no template; “genomic DNA" is from mouse B cells. The *hprt* cDNA segment is ∼250 bp, the nth1 segment is 391 bp, and the *ogg1* segment is 355 bp.

Ogg1 and Nth1 are ubiquitously expressed, and Ogg1 mRNA has been shown to be expressed in both germinal center and non-germinal center splenic B cells [12]. We performed RT-PCR and confirmed that both Ogg1 and Nth1 mRNAs are expressed in splenic B cells induced to undergo CSR to IgG3, IgG2a, or IgA in culture (Fig. 1).

**Figure 2:**
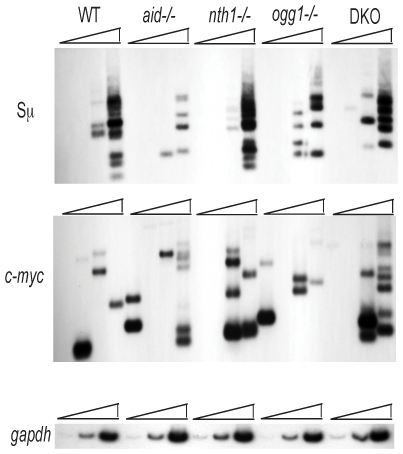
LM-PCR assay demonstrates that DSBs in the IgH Sμ region and *c-myc* gene are not detectably affected by deletion of *nth1* and/or or *ogg1* genes in splenic B cells induced to undergo CSR. The upper panel shows Sμ DSBs and the middle panel shows DSBs in the first intron of the *c-myc* gene in splenic B cells induced to switch to IgG1 for 2 days. Three-fold dose titrations of DNA were assayed (see Methods). The *gapdh* gene is amplified (lowest panel) as an internal control for template loading. The amounts used for the *gapdh* assay correspond to the 3 lowest doses of input DNA that were used for the LM-PCR assays. Similar results were obtained in two other experiments for Sμ and one other for *c-myc* DSBs.

To examine whether Ogg1 or Nth1 affect IgH S region DSBs or *c-myc* DSBs in B cells induced to undergo CSR, we used ligation-mediated PCR (LM-PCR) to assay DSBs in the IgH Sμ region, the donor S region which is involved in initial CSR events, and also in the *c-myc* locus. The breaks in Sμ are greatly reduced in *aid^−/−^* B cells, consistent with previous data indicating the dependence of Sμ DSBs upon AID, uracil DNA glycosylase, AP endonuclease 1 and 2, and mismatch repair [13], [14], [15], [16], [17], [18]. However, we did not observe any consistent change in DSB frequencies in the Sμ region or *c-myc* locus in *ogg1* ^−/−^, *nth1* ^−/−^ or double-knock out (DKO) cells relative to wild-type (WT) splenic B cells (Fig. 2). We conclude that these glycosylases do not make a substantial contribution to the induction of DSBs in the IgH Sμ region or in the *c-myc* locus. Although *c-myc* mutations and *c-myc-IgH* translocations have been shown to be dependent upon AID [2], [4], we could not detect a consistent reduction in *c-myc* DSBs in *aid^−/−^* cells by LM-PCR. This could be explained by the fact that AID-induced mutations in *c-myc* occur at very low levels, except in cells deficient in the DNA repair proteins UNG and Msh2, indicating that most AID-induced lesions in the *c-myc* gene are correctly repaired in cells with intact repair systems [2].

**Figure 3:**
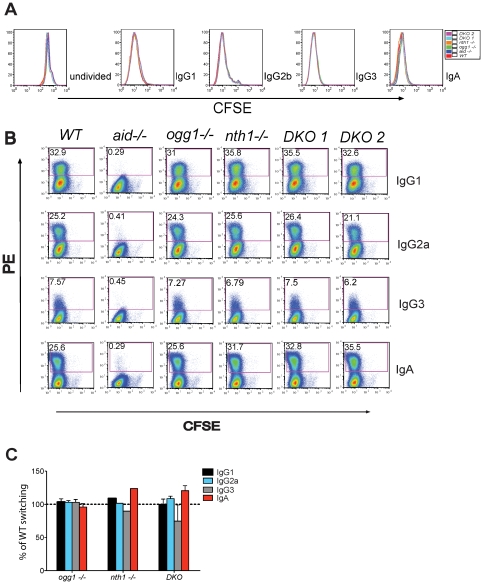
Ig class switching and proliferation of splenic B cells are not reduced in glycosylase-deficient mice. (A) Splenic B cell proliferation as assayed by dilution of CFSE assayed ∼44 hrs after treatment to switch to the indicated isotypes. (B) Flow cytometry results showing surface isotype expression (Y-axis) vs CFSE fluorescence (X axis) in cells induced to switch to the indicated isotypes for ∼44 hr. The % of the cells expressing the switched isotype are indicated within the gates. (C) Summaries of the flow cytometry data normalized to WT CSR in each experiment. The numbers of mice used are WT, 5 mice; *aid* ^−/−^, 1; *ogg1* ^−/−^, 5; *nth1* ^−/−^ 1, DKO, 2.

As another approach to determine whether Nth1 and/or Ogg1 generate DNA breaks that contribute to CSR, we assayed CSR in *ogg1* ^−/−^, *nth1* ^−/−^ or DKO splenic B cells activated to switch in culture. As CSR is dependent on cell proliferation, we first tested whether splenic B cells from mice deficient in Ogg1 or Nth1 or both (DKO) proliferated as well as wild-type (WT) littermate B cells when induced to undergo CSR in culture with LPS and other activators that induce isotype specific CSR (see Methods). As shown in Fig. 3A, proliferation as assayed by dilution of CFSE dye ∼44 hrs after initiation of the B cell cultures, was equivalent between the WT and mutant B cells. At this same timepoint, we assayed CSR to several different isotypes by staining of surface Ig expression, but found no consistent differences in IgG1, IgG3 or IgG2a CSR, although there was a slight increase in IgA CSR in *nth1^−/−^* and DKO cells (Fig. 3B, C). Fig. 3C summarizes the CSR results in the mutant B cells as a percent of WT CSR.

## Discussion

As ROS are induced in splenic B cells induced to undergo CSR in culture by LPS-treatment, we hypothesized that oxidized DNA bases would be generated during CSR, and the glycosylases Ogg1 and Nth1 would be important for their repair. Further, since repair of oxidized bases by these base excision repair enzymes involves the formation of single-strand break intermediates, we hypothesized that B cells deficient in these enzymes would show decreased DNA DSBs, and perhaps reduced CSR. However, deletion of the genes for Ogg1 and Nth1 has no consistent effect on the frequency of DSBs in the Ig Sμ region or in the *c-myc* gene or on CSR efficiency in these cells. Our results are consistent with a previous report that Ogg1 does not contribute to somatic hypermutation of antibody variable regions, another process initiated by AID, but one that does not involve formation of DNA DSBs [12]. Furthermore, another protein involved in repair of oxidized dAs, alkyladenine DNA glycosylase, has also been shown to not be involved in CSR [19]. However, as glycosylases have partially redundant functions, for example [20], it is possible that if the mice were deficient in other combinations of glycosylases, a CSR phenotype might be observed.

Previously, we found that p53-deficient LPS-activated splenic B cells have increased levels of ROS relative to WT cells, increased CSR to IgG2a, and also increased mutations at recombination junctions between Sμ and Sγ3 in IgG3-expressing cells [9]. One possible interpretation of these data was that ROS-instigated DNA breaks might contribute to CSR and S region mutations and DSBs. As no CSR is observed in the absence of AID and the deamination reaction performed by AID is an oxidation reaction, we prefer the interpretation that ROS actually stimulated the deamination activity of AID itself, thereby increasing mutations and DNA breaks. However, due to the redundancy among enzymes that repair oxidized bases, our current results do not rule out the possibility that ROS contribute to DNA breaks in Sμ and *c-myc*.

## Methods

### Mice

Both the *ogg1^−/−^* [10] and *nth1^−/−^* [11] mice were back-crossed for 8 generations to C57BL/6 mice prior to use in these experiments. Mice were housed in the Institutional Animal Care and Use Committee-approved specific pathogen-free facility at the University of Massachusetts Medical School. The mice were bred and used according to the guidelines from the University of Massachusetts Animal Care and Use Committee, Assurance number A-3306-01. Experiments are performed using spleens taken from mice sacrificed by CO2 asphyxiation followed by bilateral pneumothorax. Nth1 mice were typed using the published primers [11]. Ogg1 mice were typed using the following primers: CH597, 5′ primer for *ogg1* wt GTACCGTGCCCGCTATGTA; CH598, 3′ primer for *ogg1* wt CCTCTCGTACGCTCAGTGT; CH593, 5′ primer for *ogg1* ko, CCTTTAGGCGTCCTTTCAGTGT; CH218, 3′ primer from 3′ end of *neo* gene, GCAGCGCATCGCCTTCTAT (C. Hollander, NIH, unpublished).

### Splenic B cell cultures and analysis of CSR

Splenic B cells were isolated and induced to undergo CSR as previously described [9], except we add LPS at 25 µg/ml, anti-IgD dextran at 25 ng/ml, and human BLyS at 50 ng/ml for all CSR cultures. Also, our treatment for inducing switching to IgA has been changed to retinoic acid (10 nM), LPS (25 µg/ml), TGFβ (2 ng/ml), IL-5 (30 ng/ml), anti-IgD dextran (25 ng/ml), and human BLyS (50 ng/ml). Retinoic acid was shown to increase IgA CSR [21], and we have found that it specifically increases IgA CSR and no other isotype (unpublished).

### RT-PCR assays

cDNAs prepared by oligo dT priming from WT splenic B cells after 2 days of activation in culture to switch to IgG3, IgG2a, or IgA were amplified using the *ogg1* primers: OGG1F3, AGTTGGGCCTGGGGTACCGT and OGG1R3, GCTTGGGCCCAGCCAGCATA; and *nth1* primers: NTH1F, CGCGACTAGGATCGCGTCGG and NTH1R, GGCCCGGAGCCGTTGCATAG. *Hprt* primers were previously published [9].

### LM-PCR

LM-PCR was performed for analysis of Sμ DSBS as previously described [9]. LM-PCR for *c-myc* DSBs was similarly performed except the gene specific primer for *c-myc* (MYC3.1) (located between exons 1 and 2) was: GGGGAGGGGGTGTCAAATAATAAGA and the probe used for the hybridizing the Southern blot (MYC3-probe) was: GCAGCGATTCAGCACTGGGTGCAGG.
